# Rooting for Success: The Role of Microorganisms in Promoting Growth and Resilience in Black Alder Seedlings

**DOI:** 10.1111/1758-2229.70060

**Published:** 2024-12-06

**Authors:** Greta Striganavičiūtė, Dorotėja Vaitiekūnaitė, Milana Šilanskienė, Vaida Sirgedaitė‐Šėžienė

**Affiliations:** ^1^ Laboratory of Forest Plant Biotechnology Institute of Forestry, Lithuanian Research Centre for Agriculture and Forestry Kaunas Lithuania

**Keywords:** *Alnus glutinosa*, environmental bioremediation, phytoremediation, plant‐microbe interactions, *Pseudomonas putida*, *Rhodotorula sphaerocarpa*, *Sphingobium yanoikuyae*

## Abstract

Polycyclic aromatic hydrocarbons (PAHs) pose a global environmental risk, impacting human health. Enhancing phytoremediation with microbial‐plant interactions could help mitigate these pollutants. However, tree responses to PAHs are unclear, necessitating controlled studies before field experiments. This study examined how PAH‐degrading microbes affect black alder ( *Alnus glutinosa* L.) seedlings grown hydroponically, hypothesizing that specific microbes improve growth and stress tolerance. Two half‐sib families (41–65–7 K, 13–99–1 K) were inoculated with *Rhodotorula sphaerocarpa* (*R.s*.), *Pseudomonas putida* (*P.p.*), and *Sphingobium yanoikuyae* (*S.y*.). Results showed family‐dependent and microbe‐specific effects, with family 41–65–7 K showing enhanced shoot growth (threefold increase by *R.s.*) and higher carotenoid levels. Antioxidant enzyme activities varied: *R.s.* elevated superoxide dismutase activity by 4.8‐fold in 13–99–1 K, while catalase activity increased but decreased in 41–65–7 K. Principal component analysis revealed distinct phytochemical clustering based on microbial treatment, highlighting genotype‐specific modulations. Each microorganism had unique plant growth‐promoting traits, with *P.p.* producing the most phytohormone and *S.y.* fixing nitrogen. These findings support targeted microbial inoculation for effective remediation of PAH‐contaminated environments.

## Graphical abstract

Microbial inoculation of black alder seeds with PAH‐degrading strains affects growth and biochemical responses. The 41–65–7 K half‐sib family exhibited increases in shoot growth and root length, particularly with *Rhodotorula sphaerocarpa*. The study identified varying effects on chlorophyll ratios, antioxidant enzyme activities, and stress responses across genotypes, indicating the role of specific microorganisms in influencing plant resilience and growth.

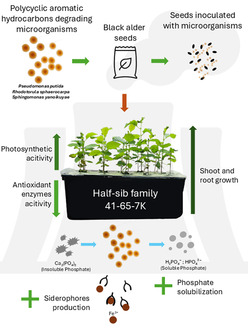

## 1 Introduction

Polycyclic aromatic hydrocarbons (PAHs) are a group of persistent, environmentally ubiquitous organic compounds that are known to have toxic effects on organisms, primarily through interference with cellular membrane functions and associated enzyme systems (Abdel‐Shafy and Mansour 2016). Incomplete combustion is their main source (Latimer and Zheng 2003). PAHs can enter plants either through their leaves or through the soil (Ailijiang et al. 2022; Houshani et al. 2019). However, soil serves as a storage and transfer station and bears more than 90% of the environmental load of these compounds (Cao et al. 2020).

Remediating PAH‐contaminated environments is crucial to mitigate the ecological and human health risks associated with these pollutants. Out of several remediation strategies, phytoremediation stands out as a sustainable low‐cost method that uses plants to absorb, degrade, or stabilise environmental contaminants (Jacob et al. 2018; Van Aken, Correa, and Schnoor 2010; Wuana and Okieimen 2011).

Black alder ( *Alnus glutinosa* L.) is a versatile widespread species increasingly used in phytoremediation efforts due to its substantial ecological benefits (Amirbekov et al. 2024; Fuller, Germaine, and Rathore 2023). Previously alders were linked with mineral oil hydrocarbons, phenol, and PAH degradation studies and used to reclaim former industrial sites (Frick, Farrell, and Germida 2000; Józefowska, Woś, and Pietrzykowski 2016; Plamping et al. 2017; Roy, Khasa, and Greer 2011).

Due to their rapid adaptability, microorganisms have been extensively used to degrade or remediate environmental hazards (Ghosal et al. 2016). Certain bacteria, such as *Pseudomonas* spp. and *Sphingobium* sp., can degrade PAHs through enzymatic processes, breaking them down into less harmful compounds (Premnath et al. 2021). This study focuses on three PAH‐degrading microorganisms: *Rhodotorula sphaerocarpa* (S.Y. Newell and Fell) Q.M. Wang, F.Y. Bai, M. Groenewald and Boekhout (*R.s*.), *Pseudomonas putida* Trevisan (*P.p*.), and *Sphingobium yanoikuyae* Yabuuchi et al. (*S.y*.). Different strains of *Rhodotorula, Sphingobium* and *Pseudomonas* have been shown to degrade PAHs enzymatically (Mandal and Das 2017). Understanding the phytoremediation potential as well as the plant growth promoting traits (PGPTs) of these microorganisms is crucial for advancing bioremediation strategies aimed at mitigating PAH contamination. Microorganism PGPTs encompass a range of mechanisms that contribute to improved plant health and increased tolerance to environmental stressors (da Costa et al. 2014; Hayat et al. 2010; Mohamed et al. 2022). These include phytohormone production, capabilities to enhance nutrient assimilation, biofilm formation for easier colonisation, and so forth (Aasfar et al. 2021; Limtong and Koowadjanakul 2012; Pang et al. 2024; Ruanpanun et al. 2010; Srivastava 2023). Understanding and harnessing these PGPTs can significantly bolster phytoremediation outcomes, making them more efficient and sustainable in remediating contaminated environments as can be seen from previous studies (Bruno et al. 2021; Silambarasan et al. 2020).

Thus, this study aims to investigate two primary objectives related to the interactions between microorganisms and black alder ( *Alnus glutinosa* ) seedlings in the context of environmental bioremediation. Firstly, it seeks to assess the impact of PAH‐degrading bacteria on the growth parameters and antioxidant enzyme activities of black alder seedlings. Secondly, the study aims to determine the PGPTs of the tested microorganisms. By evaluating these objectives, the research aims to enhance the understanding of how specific microorganisms could potentially aid in both phytoremediation efforts and the sustainable management of PAH‐contaminated environments.

## 2 Materials and Methods

During the study, seeds of two half‐sib families (individuals sharing one parent) of black alder were inoculated with three PAH‐degrading microorganism strains, then germinated in rockwool cubes, and subsequently cultivated under hydroponic conditions. Later, growth parameters were measured, and biochemical parameters were evaluated. Additionally, the microorganisms were assessed for plant growth‐promoting traits. Black alder seeds were harvested from trees in a seed plantation managed by the Šiauliai City Forest Department (Lithuania) in 2021. Two half‐sib families, 41–65–7 K and 13–99–1 K, were chosen for the experiment due to their demonstrated resilience to various PAHs in prior studies (Striganavičiūtė et al. 2024).

### 2.1 PAH‐Degrading Microorganisms

Three PAH‐degrading microorganisms were selected for the experiment based on their non‐toxicity to humans and plants, as well as their ability to colonise plant tissues: *Pseudomonas putida* Trevisan (*P.p*.)—DSM‐No. 28064, *Sphingobium yanoikuyae* Yabuuchi et al. (*S.y*.)—DSM‐No. 6900 (both acquired from The Leibniz Institute DSMZ), and *Rhodotorula sphaerocarpa* (S.Y. Newell and Fell) Q.M. Wang, F.Y. Bai, M. Groenewald and Boekhout (*R.s*.)—MUCL No.‐030605 (obtained from the Belgian Co‐ordinated Collections of Micro‐organisms BCCM).

### 2.2 Seed Inoculation With PAH‐Degrading Microorganisms and Plant Preparation for the Experiment

To inoculate black alder seeds, the microorganisms were separately cultured in liquid LB medium (Duchefa Biochemie, Haarlem, the Netherlands) at pH 7 for 3 days in a thermal shaker at 30°C and 1.33 × g. After incubation, 10 mL of each culture was centrifuged at 3500 × g for 5 min to pellet the cells. The supernatant was discarded, and the cells were resuspended in 10 mL of 0.9% NaCl solution. This washing step was repeated three times with fresh 0.9% NaCl solution. Subsequently, the optical density (OD_600_) of each microorganism solution was adjusted to 1 using a SpectroStar Nano microplate reader (BMG Labtech, Offenburg, Germany).

Next, 500 black alder seeds per family were soaked in 20 mL of the adjusted microorganism solution for 30 min, with intermittent mixing. Control groups were soaked in 20 mL of 0.9% NaCl solution. After soaking, the seeds were air‐dried and then planted into rockwool cubes. Twenty‐four hours prior to sowing, the rockwool cubes were soaked in distilled water adjusted to pH 5.6, using 0.1 M NaOH and 0.1 M HCl solutions (from Duchefa Biochemie, Haarlem, the Netherlands). After soaking, excess water was drained, and the cubes were irrigated with modified full‐strength (100%) Hoagland's nutrient solution, also adjusted to pH 5.6, before planting the seeds. This modified nutrient solution composition included 6 mM KNO_3_, 2.32 mM Ca(NO_3_)_2_ × 4 H_2_O, 1.86 mM MgSO_4_ × 7 H_2_O, 1 mM NH_4_H_2_PO_4_, 46 μM H_3_BO_3_, 9 μM MnCl_2_ × 4H_2_O, 8.99 μM C_12_H_12_Fe_2_O_18_, 0.76 μM ZnSO_4_ × 7 H_2_O, 0.5 μM CuSO_4_ × 5 H_2_O, and 0.58 μM Na_2_MoO_4_ × 2 H_2_O.

The trays containing the rockwool cubes with sowed seeds were covered with plastic lids and kept in darkness for 1 week at a day/night temperature of 25°C/20°C. Subsequently, the trays were transferred to an environment with white light, providing an irradiance of 94.5 μmol m^−2^ s^−1^, with the covers removed, and incubated for 4 weeks until the seedlings reached the four‐true‐leaf stage (growth stage BBCH14, as per Guilayn et al. (2020)). Throughout this period, seedlings were watered as needed with half‐strength (50%) Hoagland's nutrient solution.

### 2.3 Hydroponic Setup and Experimental Conditions

Black plastic containers, each with a volume of 10 L and dimensions of 30 × 25 × 12 cm, were covered with 2 cm thick polystyrene foam boards. A plastic netting panel with 20 holes was custom fitted to accommodate the rockwool cubes.

Air pumps (Union Star Air AC‐500, 230 V, 50 Hz, 2 W, China) connected to plastic air hoses, non‐return valves (12 × 7 × 3 cm), and 2 cm air pebbles supplied constant aeration to the containers. The seedlings were grown for 4 weeks under controlled conditions in a growth chamber maintained at 25°C/20°C (day/night) with a 16/8 h photoperiod of white light (irradiance 94.5 μmol m^−2^ s^−2^).

After 4 weeks, morphometric parameters including shoot and root length were measured. Mean shoot length was calculated at the beginning and end of the experiment to assess growth. Root length was measured only at the end of the experiment. Leaves were harvested for biochemical analyses.

### 2.4 Biochemical Analysis

#### 2.4.1 Preparation of Extracts for Photosynthetic Pigment, Secondary Metabolite, Malondialdehyde (MDA), and Sugar Determination

Collected leaves (3 × 0.1 g per group) were pulverised using a “Precellys 24” tissue homogeniser (Bertin Technologies, Montigny‐le‐Bretonneux, France) at 1956 × *g* for 30 s with two metal balls. Subsequently, 1.5 mL of 80% ethanol (v/v in water, MV GROUP Production, Lithuania) was added, and the mixture was homogenised again at 1956 × *g* for 30 s. The samples were then centrifuged at 21,910 × *g* for 30 min at 4°C using a Hettich Universal 32R centrifuge (Andreas Hettich GmbH and Co. KG, Tuttlingen, Germany). The resulting supernatant was collected and used for chlorophyll, carotenoid, secondary metabolite, malondialdehyde, and soluble sugar analysis.

#### 2.4.2 Measurement of Total Phenolic Content (TPC)

The determination of total phenolic content (TPC) utilised a modified Lowry method with Folin–Ciocalteu reagent. Specifically, the extract was mixed with Folin–Ciocalteu reagent (1:9 w/v in water). After a 5‐min incubation, Na_2_CO_3_ was added. The mixture was then kept in the dark for 1 h, and the absorbance of the sample was measured at 725 nm using a microplate reader. For a detailed protocol, please refer to Čėsnienė et al. (2023) publication.

#### 2.4.3 Measurement of Total Flavonoid Content (TFC)

The determination of total flavonoid content (TFC) was done following the method described by Chang et al. (2020). Absorbance measurements were conducted at 415 nm. Extract was mixed with reaction buffer containing absolute ethyl alcohol (MERCK, Germany), aluminium chloride solution (Alfa Aesar, Germany), potassium acetate (Sigma Aldrich, USA), and distilled water. For detailed procedures and calculations, please refer to the methodology outlined in the publication by Čėsnienė et al. (2023).

#### 2.4.4 Measurement of Chlorophyll *a* and *b*, and Total Carotenoids

Absorption levels of the extract were measured at wavelengths of 470, 648 and 664 nm using a SpectroStar Nano microplate reader (BMG Labtech, Offenburg, Germany) and 96‐well microplates. Detailed formulas and models for analysis were utilised as described in Čėsnienė et al. (2023).

#### 2.4.5 Measurement of Malondialdehyde (MDA)

MDA analysis involved mixing the supernatant with reaction mixture, prepared by combining tri‐chloroacetic acid (Molar chemicals Kft, Hungary) and thiobarbituric acid (Alfa Aesar, Germany) following the method described in Čėsnienė et al. (2023). The resulting mixture was then incubated at 95°C for 30 min and subsequently chilled on ice. Absorbance of the supernatant was measured at 440, 532, and 600 nm.

#### 2.4.6 Measurement of Soluble Sugars (SS)

Soluble sugar (SS) determination involved mixing the sample with anthrone reagent (Carl Roth, Germany). The anthrone reagent was prepared by combining 0.1 g of anthrone with 100 mL of concentrated H_2_SO_4_ (Chempur, Poland), following the method detailed in Čėsnienė et al. (2023). The mixture was then maintained at 90°C for 1 h, and the absorbance was measured at 620 nm.

#### 2.4.7 Preparation of Potassium Phosphate Buffer

To assess the activity of antioxidant enzymes, K‐phosphate buffers with varying pH levels were essential. These buffers were formulated by mixing two stock solutions: one containing 1 M K_2_HPO_4_ (Carl Roth, Germany) and the other 1 M KH_2_PO_4_ (Chempur, Poland). The experimental conditions required K‐phosphate buffers with pH values of 6.5, 7, and 7.8.

#### 2.4.8 Preparation of Extracts for Antioxidant Enzyme Assays

The preparation of extracts for enzyme analyses follows the methods outlined in Čėsnienė et al. (2023). Briefly, the fresh biomass (3 × 0.1 g per group) was ground using liquid nitrogen and mixed with an extraction buffer containing K‐phosphate buffer (pH 7.8), Triton‐X, polyvinylpolypyrrolidone (PVPP), and ascorbic acid (ASC). After centrifugation at 21,910 × *g* and −4°C for 1 h, the supernatant was used for analysing total protein (PROT) levels, catalase (CAT), and superoxide dismutase (SOD) activity.

For ascorbate peroxidase (APX), guaiacol peroxidase (POX), glutathione reductase (GR), and glutathione S‐transferase (GST) analysis, the supernatant was further purified using Sephadex G‐25 columns (Column PD‐10, Cytiva, Gillingham, UK) (Kozlowski, Buchala, and Métraux 1999). The filtered bulk (non‐fractioned) extract was used further. All extraction procedures were performed on ice to preserve sample integrity (Singleton, Orthofer, and Lamuela‐Raventós 1999).

#### 2.4.9 Total Protein (PROT) Quantification

The concentration of PROT was determined Čėsnienė et al. (2023). Briefly, crude extract was mixed with Biuret reagent containing CuSO_4_, Na‐K tartrate, Na_2_CO_3_ in NaOH solution, and Folin–Ciocalteau reagent. Following incubation at room temperature, absorbance was measured at 660 nm. Protein concentrations were expressed as micrograms of Bovine Serum Albumin (BSA) equivalent per millilitre of crude extract.

#### 2.4.10 Catalase Enzyme (CAT) Activity Measurement

The activity of the catalase enzyme (CAT) was assessed (Čėsnienė et al. 2023). For the assay, a portion of the extract used for antioxidant enzyme testing was mixed with K‐phosphate buffer (pH 7) and H_2_O_2_ solution. Absorbance readings were taken at regular intervals.

#### 2.4.11 Superoxide Dismutase Enzyme (SOD) Activity Measurement

SOD activity was assessed by mixing the extract with reaction buffer composed of K‐phosphate buffer (pH 7.8), methionine, nitro blue tetrazolium (NBT), ethylenediamine tetraacetic acid (EDTA), and riboflavin. The reaction mixture was exposed to white light (irradiance 30 μmol m^−2^ s^−2^) until the samples darkened compared to the control group. Absorbance was then measured at 550 nm to determine SOD activity (Čėsnienė et al. 2023).

#### 2.4.12 Guaiacol Peroxidase Enzyme (POX) Activity Measurement

For POX assays, a portion of the filtered extract was mixed with a reaction solution consisting of pyrogallol dissolved in a 50 mM K‐phosphate buffer (pH 6.5) and 10% H_2_O_2_. The activity of POX was then assessed at 430 nm, monitoring the kinetic change over time at intervals. (Čėsnienė et al. 2023).

#### 2.4.13 Ascorbate Peroxidase Enzyme (APX) Activity Measurement

The method described in Čėsnienė et al. (2023) was employed for APX assays, where filtered extract was combined with ASC solution and 10% H_2_O_2_ solution. APX activity was monitored at 290 nm, assessing the decrease in absorbance over time at intervals.

#### 2.4.14 Glutathione S‐Transferase Enzyme (GST) Activity Measurement

GST activity was assessed by combining the extract with a reaction buffer containing CDNB and GSH. Absorbance at 340 nm was measured at regular intervals, and enzyme activity was calculated based on the change in absorbance over time, following the method outlined by Striganavičiūtė et al. (2024).

#### 2.4.15 Glutathione Reductase Enzyme (GR) Activity Measurement

To measure GR enzyme activity, the extract was mixed with a reaction buffer consisting of HEPES buffer (pH 8), EDTA, and NADPH. After the addition of GSSG, changes in absorbance at 340 nm were monitored over time Čėsnienė et al. (2023).

### 2.5 Plant Growth Promoting Trait Assessment

To identify the traits in tested microorganisms that could enhance plant growth, a qualitative in vitro PGPT assessment was conducted on selective media. Each PGPT was screened in independent biological triplicates using fresh colonies for each iteration. The microorganisms were cultured at a temperature of 25°C ± 1°C.

#### 2.5.1 Siderophore Production

For the experiment, freshly prepared Chromeazurol S (CAS) reagent was mixed with LB agar medium in a 1:9 ratio, as both bacteria and yeast thrived on this medium in preliminary tests. The isolates were spot inoculated onto the medium and incubated for up to 1 week. Siderophore production was indicated by the appearance of orange or yellow zones surrounding the colonies Schwyn and Neilands (1987).

#### 2.5.2 Potassium Solubilisation

To evaluate potassium solubilisation (Fatharani and Rahayu 2018; Setiawati and Mutmainnah 2016), Aleksandrow agar medium (HiMedia, Mumbai, India) was used, containing feldspar powder as the sole and insoluble potassium source. The isolates were inoculated onto the medium and incubated for up to 7 days. Potassium solubilisation was indicated by the formation of clear zones around the colonies.

#### 2.5.3 Nitrogen Fixation

To screen for potential nitrogen fixation, nitrogen‐free Jensen's medium (HiMedia, India) was used following the method of Jasim et al. (2015). Microorganism samples were spot inoculated onto the medium, with one sample per plate, and incubated for up to 1 week. Colony growth was then assessed, and those exhibiting well‐defined growth were identified as putative diazotrophs.

#### 2.5.4 Indole‐3‐Acetic Acid Production

To determine if the microorganisms could produce indole‐3‐acetic acid (IAA) in a tryptophan‐dependent manner, a modified Salkowski reagent test was conducted (Gordon and Weber 1951). The microorganisms were cultured in LB broth (Duchefa Biochemie) supplemented with 0.15% (w/v) tryptophan in a thermal shaker at 90 rpm and 25°C for 24 h in the dark. After incubation, 1.5 mL of the suspension was transferred to a microtube and centrifuged at 16,300 × *g* for 5 min. Then, 150 μL of the supernatant was mixed with an equal volume of Salkowski reagent (consisting of 1 mL of 0.5 M FeCl_3_ and 49 mL of 35% HClO_4_ v/v) in a 96‐well microplate. The microplate was then incubated in the dark for 30 min, after which the optical density was measured at 530 nm using a Synergy HT Multi‐Mode Microplate Reader.

#### 2.5.5 Biofilm Production

Briefly, the ability of microorganisms to form biofilms was assessed using a modified tissue culture plate method (Mohamed et al. 2016). Isolates were grown overnight in liquid LB. The following day, 2 μL of this suspension was added to each well of a sterile, 96‐well tissue culture plate, followed by 198 μL of LB medium supplemented with 1% glucose. Wells containing only 200 μL of LB medium with 1% glucose served as controls. The plate was incubated overnight. After incubation, the wells were washed three times with sterile water and left to air dry. The biofilm layer was stained with 0.1% Gentian violet solution for 15 min, then washed and air‐dried again. The stained biofilm was then solubilised in 95% ethanol for 30 min. Optical density (OD) was measured at 630 nm using a Synergy HT Multi‐Mode Microplate Reader, with 95% ethanol serving as the control. The optical density cut‐off (ODc) was calculated as the average OD of the control plus three times the standard deviation of the control. Biofilm formation was classified as weak (approximately ODc), moderate (2–4 times ODc), or strong (more than four times ODc).

#### 2.5.6 Phosphate Assimilation

Microorganism isolates were evaluated for their ability to solubilise and mineralize phosphate Chen and Liu (2019). Two different phosphate sources were used: tricalcium phosphate for testing inorganic phosphate solubilisation, and soy lecithin for assessing organic phosphate mineralization. The microorganisms were spot inoculated onto the respective media and incubated for up to 1 week. Successful phosphate solubilisation and/or mineralization was indicated by the presence of clear zones around the colonies.

### 2.6 Statistical Analysis

The findings were compiled, and graphs were generated using Microsoft Office Excel. Statistical analyses were conducted using SPSS, version 28.0.1.1 (IBM Inc.), employing the Kruskal–Wallis *H* test for independent samples as a non‐parametric alternative to one‐way ANOVA. Further pairwise comparison on ranks was done with Dunn's test (*p* < 0.05). The confidence levels were 95%. Pairwise comparisons from Dunn's test were reported with adjusted (Bonferroni correction) *p*‐values to indicate which specific groups differed significantly.

Principal Component Analysis (PCA) was employed to investigate how different microorganisms (*P.p*., *S.y*., and *R.s*.) affected chlorophyll a/b ratio and antioxidant enzyme activity, TFC, TPC, SS, MDA, and carotenoid concentration in two half‐sib families. Data preparation included addressing missing values through omission, ensuring complete datasets for robust analysis. Numeric variables were standardised to unit variance and centred using the ‘scale()’ function in R (version 4.3.1, R Foundation for Statistical Computing, [Vienna, Austria]) to ensure equal contribution of each variable to the PCA. Four separate PCA analyses were conducted for the control, *P.p*., *S.y*., and *R.s*. treatments within each half‐sib family. The ‘prcomp()’ function computed principal components based on the correlation matrix of the standardised variables. The variance explained by each principal component was visualised through scree plots, aiding in identifying significant components. Scatter plots of individuals and variables projected onto the principal component space were generated to illustrate the relationships and effects of each microorganism within each family. Visualisation was enhanced using the ‘factoextra’ and ‘ggplot2’ packages in R.

## 3 Results

### 3.1 Effects of Microbial Inoculation on Black Alder Growth Parameters

**FIGURE 1:**
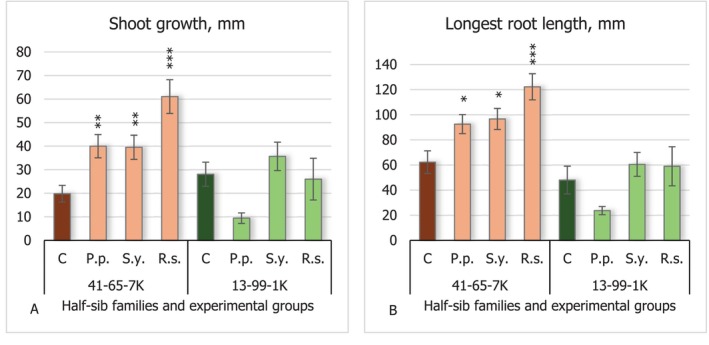
Comparison of shoot growth (mm) (A) and longest root length (B) in black alder half‐sib families (41–65–7 K and 13–99–1 K) across different bacterial treatments: Control (C), *Pseudomonas putida* (*P.p*.), *Sphingobium yanoikuyae* (*S.y*.), and *Rhodotorula sphaerocarpa* (*R.s*.). The graphs use standard error (SE) bars. Statistically significant differences from the control group were determined by Kruskal–Wallis H test: *p < 0.05; **p < 0.01; ***p < 0.001.

During the study, the impact of PAH‐degrading bacteria on black alder seedlings' shoot growth (Figure 1A) and longest root length (Figure 1B) was measured. The results showed that none of the bacteria had a significant impact on these morphological parameters in the seedlings of the half‐sib family 13–99–1 K. Even so, *Pseudomonas putida* (*P.p*.) expressed a tendency to lower these metrics. In contrast, the seedlings of the 41–65–7 K half‐sib family showed positive effects, with all tested bacteria increasing these growth parameters, with yeast *Rhodotorula sphaerocarpa* (*R.s*.) making the biggest impact: A threefold increase in shoot growth and twofold increase in longest root length (*p* < 0.001).

#### 3.1.1 Effects of Microbial Inoculation on Black Alder Biochemistry

**FIGURE 2:**
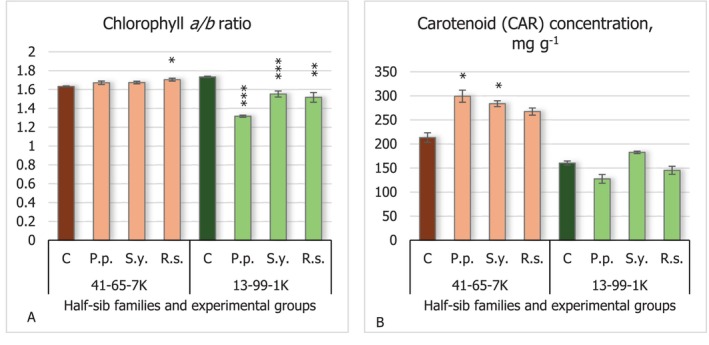
Comparison of chlorophyll *a/b* ratio (A) and carotenoid concentration (B) in black alder half‐sib families (41–65–7 K and 13–99–1 K) across different bacterial treatments: Control (C), *Pseudomonas putida* (*P.p*.), *Sphingobium yanoikuyae* (*S.y*.), and *Rhodotorula sphaerocarpa* (*R.s*.). The graphs use standard error (SE) bars. Statistically significant differences from the control group were determined by Kruskal–Wallis H test: *p < 0.05; **p < 0.01; ***p < 0.001.

Chlorophyll *a/b* ratio results showed different reactions of half‐sib families to inoculation with microorganisms (Figure 2A). *R.s*. increased the chlorophyll *a/b* ratio in the seedlings of the 41–65–7 K half‐sib family (*p* < 0.05). In contrast, the seedlings of the 13–99–1 K half‐sib family reacted oppositely, with all bacterial treatments decreasing the chlorophyll *a/b* ratio compared with the control groups (*p* < 0.01 for *R.s*. and *p* < 0.001 for *P.p*. and *S.y*.). The biggest decrease in the chlorophyll *a/b* ratio was noted under the effect of *Pseudomonas putida* (*P.p*.). Interestingly, it can be observed that *P.p*. also exhibited a tendency to decrease the aforementioned growth parameters in the same half‐sib family (Figure 1).

The variance of carotenoid concentration (Figure 2B) showed that only the seedlings of the 41–65–7 K half‐sib family were significantly affected by the two bacteria, *P.p*. and *S.y*., both increased the carotenoid levels (*p* < 0.05).

**FIGURE 3:**
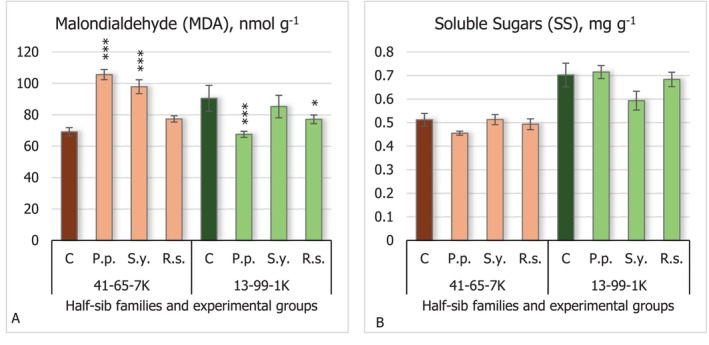
Comparison of malondialdehyde (MDA) levels (A) and soluble sugars (B) concentration in black alder half‐sib families (41–65–7 K and 13–99–1 K) across different bacterial treatments: Control (C), *Pseudomonas putida* (*P.p*.), *Sphingobium yanoikuyae* (*S.y*.), and *Rhodotorula sphaerocarpa* (*R.s*.). The graphs use standard error (SE) bars. Statistically significant differences from the control group were determined by Kruskal–Wallis H test: *p < 0.05; ***p < 0.001.

The results of malonaldehyde (MDA) levels (Figure 3A) showed different responses among the half‐sib families. Seedlings of the 41–65–7 K half‐sib family exhibited increased MDA levels when the seeds were inoculated with *P.p*. and *S.y*. bacteria (*p* < 0.001), while the seedlings of the 13–99–1 K half‐sib family showed slightly decreased MDA levels (*p* < 0.001 for *P.p*. and *p* < 0.05 for *R.s*.). Lower MDA levels indicate reduced lipid peroxidation and oxidative stress. The results on soluble sugar (SS) (Figure 3B) showed no significant differences across the experimental groups. However, it is notable that the overall soluble sugar concentration was higher in the seedlings of the 13–99–1 K half‐sib family compared to the 41–65–7 K family.

**FIGURE 4:**
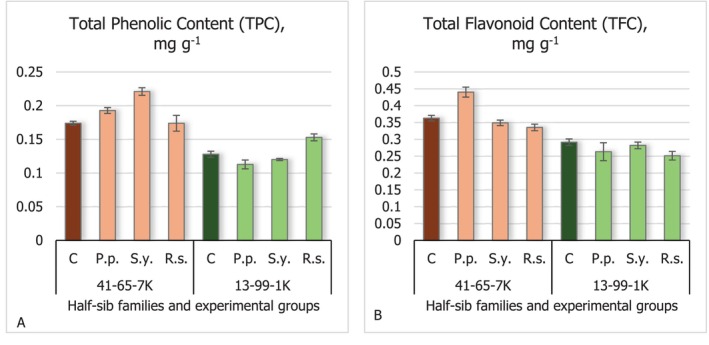
Comparison of total phenol content (TPC) (A) and total flavonoid content (TFC) (B) concentration in black alder half‐sib families (41–65–7 K and 13–99–1 K) across different bacterial treatments: Control (C), *Pseudomonas putida* (*P.p*.), *Sphingobium yanoikuyae* (*S.y*.), and *Rhodotorula sphaerocarpa* (*R.s*.). The graphs use standard error (SE) bars. Statistically significant differences from the control group were determined by Kruskal–Wallis H test: *p < 0.05; ***p < 0.001.

Results of total phenolic content (TPC) (Figure 4A) and total flavonoid content (TFC) (Figure 4B) showed no significant differences across the experimental groups. However, the seedlings of the 41–65–7 K half‐sib family exhibited higher levels of these two parameters compared to the 13–99–1 K family. The highest TPC content was observed in the seedlings of the 41–65–7 K half‐sib family inoculated with *S.y*. bacteria, while the highest TFC was noted in seedlings inoculated with *P.p*. bacteria.

**FIGURE 5:**
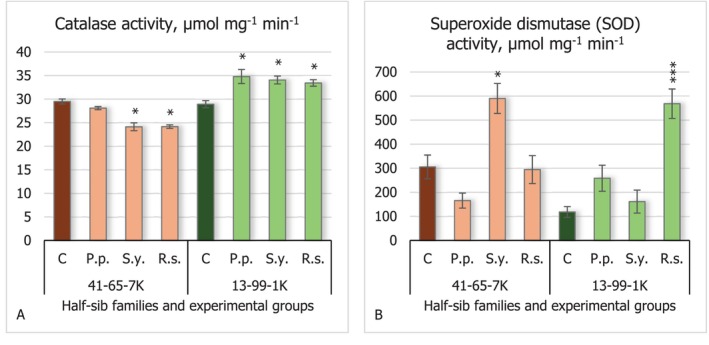
Comparison of catalase (CAT) activity (μmol mg^−1^ min^−1^) (A) and superoxide dismutase (SOD) activity (μmol mg^−1^ min^−1^) (B) in black alder half‐sib families (41–65–7 K and 13–99‐–1 K) across different bacterial treatments: Control (C), *Pseudomonas putida* (*P.p*.), *Sphingobium yanoikuyae* (*S.y*.), and *Rhodotorula sphaerocarpa* (*R.s*.). The graphs use standard error (SE) bars. Statistically significant differences from the control group were determined by Kruskal–Wallis H test: *p < 0.05; ***p < 0.001.

Regarding the results of antioxidant enzyme activity, catalase (CAT) activity (Figure 5A) in the seedlings of the 41–65–7 K half‐sib family experienced a decrease when seeds were inoculated with *S.y*. and *R.s*. strains (*p* < 0.05). Conversely, seedlings of the 13–99–1 K half‐sib family experienced increased CAT activity when seeds were inoculated with all the tested microorganisms (*p* < 0.05).

Results of superoxide dismutase (SOD) activity (Figure 5B) showed a significant increase in both black alder half‐sib families. In the seedlings of the 41–65–7 K half‐sib family, SOD activity was increased by *S.y*. (*p* < 0.05), and in the seedlings of the 13–99–1 K half‐sib family, there was a 4.8‐fold increase in SOD activity by yeast *R.s*. compared with the control group (*p* < 0.001). Additionally, SOD levels differed by 2.5‐fold between controls in these families, and seedlings from the 13–99–1 K half‐sib family exhibited lower SOD activity compared to the 41–65–7 K family.

**FIGURE 6:**
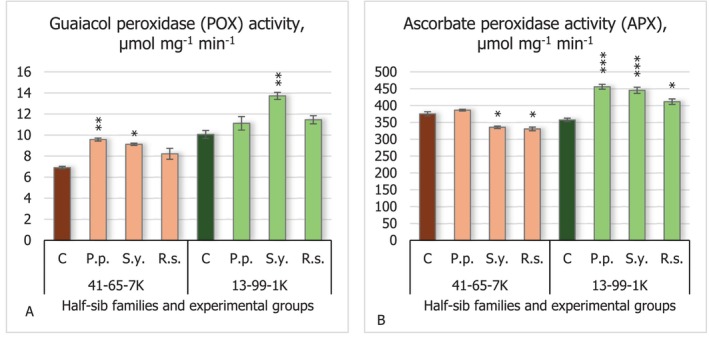
Comparison of guaiacol peroxidase (POX) activity (μmol mg^−1^ min^−1^) (A) and ascorbate peroxidase (APX) activity (μmol mg^−1^ min^−1^) (B) in black alder half‐sib families (41–65–7 K and 13–99–1 K) across different bacterial treatments: Control (C), *Pseudomonas putida* (*P.p*.), *Sphingobium yanoikuyae* (*S.y*.), and *Rhodotorula sphaerocarpa* (*R.s*.). The graphs use standard error (SE) bars. Statistically significant differences from the control group were determined by Kruskal–Wallis H test: *p < 0.05; **p < 0.01; ***p < 0.001.

The results of guaiacol peroxidase (POX) activity (Figure 6A) showed only positive effects from microorganisms by increasing POX activity. The *S.y*. strain increased POX activity in the seedlings of both tested half‐sib families (*p* < 0.05 for 41–65–7 K and *p* < 0.01 for 13–99–1 K half‐sib family seedlings), while *P.p*. bacteria increased POX activity in the seedlings of the 41–65–7 K half‐sib family (*p* < 0.01).

There were dual results in the case of ascorbate peroxidase (APX) activity (Figure 6B). Seedlings of the 41–65–7 K half‐sib family experienced a decrease in APX activity when seeds were inoculated with *S.y*. and *R.s*. microorganisms(*p* < 0.05). Conversely, seedlings of the 13–99–1 K half‐sib family experienced increased APX activity when inoculated with all the tested microorganisms: *P.p*. by 28%, *S.y*. by 25% (for both *p* < 0.001), and *R.s*. by 15% (*p* < 0.01).

**FIGURE 7:**
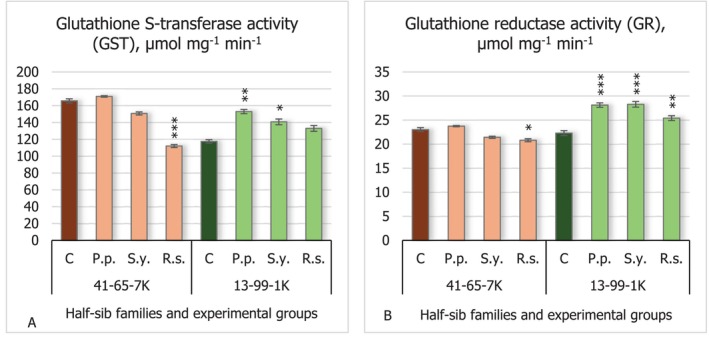
Comparison of glutathione S‐transferase activity (GST) (μmol mg^−1^ min^−1^) (A) and glutathione reductase activity (GR) (μmol mg^−1^ min^−1^) (B) in black alder half‐sib families (41–65–7 K and 13–99–1 K) across different bacterial treatments: Control (C), *Pseudomonas putida* (*P.p*.), *Sphingobium yanoikuyae* (*S.y*.), and *Rhodotorula sphaerocarpa* (*R.s*.). The graphs use standard error (SE) bars. Statistically significant differences from the control group were determined by Kruskal–Wallis H test: *p < 0.05; **p < 0.01; ***p < 0.001.

The results of glutathione S‐transferase (GST) activity (Figure 7A) showed that *R.s*. decreased its activity in the seedlings of the 41–65–7 K half‐sib family (*p* < 0.001). In contrast, the seedlings of the 13–99–1 K half‐sib family experienced increased GST activity when seeds were inoculated with *P.p*. (*p* < 0.01) and *S.y*. (*p* < 0.05) bacteria.

Results of this study showed that the *R.s*. microorganism decreased glutathione reductase (GR) activity (Figure 7B) in the seedlings of the 41–65–7 K half‐sib family (*p* < 0.05). An opposite effect of the tested microorganisms was detected in the seedlings of the 13–99–1 K half‐sib family, where all microorganisms increased GR activity (*p* < 0.001 for *P.p*. and *S.y*. and *p* < 0.01 for *R.s*.).

Overall, the study demonstrates that the effects of PAH‐degrading microorganisms on black alder seedlings are genotype‐dependent, with the 41–65–7 K half‐sib family generally showing more positive growth responses and enzyme activity changes compared to the 13–99–1 K family. *Rhodotorula sphaerocarpa* (*R.s*.) had significant higher positive effect on shoot growth and root length in the 41–65–7 K family.

#### 3.1.2 Principal Component Analysis (PCA) of Black Alder Responses to Microorganism Inoculation

Principal Component Analysis (PCA) was employed to evaluate the impact of microbial treatments on chlorophyll *a/b* ratio, MDA, carotenoid levels, total phenolic content, total flavonoid content, soluble sugars and antioxidant enzyme activity profiles.

**FIGURE 8:**
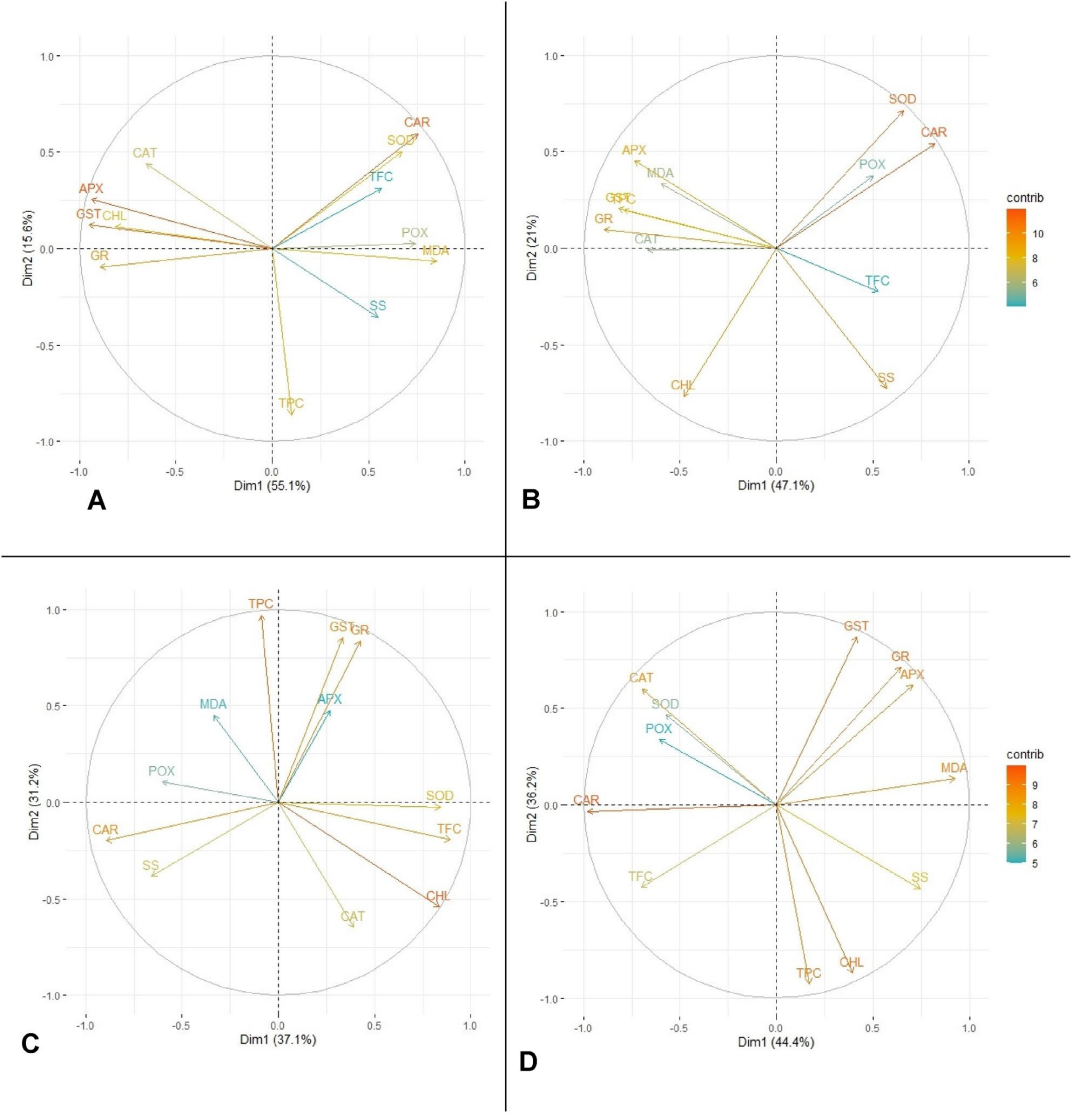
PCA results for phytochemical profile in black alder half‐sib family 41–65–7 K across different microbial treatments: Control (A), *Pseudomonas putida* (*P.p*.) (B), *Sphingobium yanoikuyae* (*S.y*.) (C), and *Rhodotorula sphaerocarpa* (*R.s*.) (D).

In all four groups of family 41–65–7 K the two principal components explain 68.1% to 80.6% of cumulative variance. In the control group (Figure 8A), Dim1 strongly positively correlates with MDA and POX (grouped together) and negatively with CHL, GST, GR, APX and CAT (grouped together). CAR and SOD correlate more so with Dim 1 than Dim2. TPC correlates strongly negatively corelates with Dim2, while TFC and SS correlate moderately with both components. This would be indicative that Dim2 can be associated with phenols, and Dim1 with mainly antioxidant enzymes, oxidative damage and chlorophyl. CAR, SOD and TFC are grouped together, indicating strong links. In the *P.p*. group (Figure 8B) Dim1 is strongly negatively linked with a group consisting of APX, MDA, GST, TPC, GR and CAT and moderately positively linked with POX, CAR and TFC. Dim2 on the other hand negatively correlates with CHL and SS, while SOD relates to both equally. SOD, CAR and POX are grouped together, while TFC, SS and CHL stand apart. This again indicates that Dim1 can be broadly linked with oxidative stress. Dim2 designation would less ambiguous but could be linked with the photosynthetic system. In group *S.y*. (Figure 8C) we can observe a comparatively more scattered distribution, though GST, GR and APX are grouped closely. Dim1 correlates positively with SOD, TFC, CHL and negatively with POX, CAR and SS. Dim2 correlates positively with TPC, GST, GR and negatively with CAT. MDA, APX contribute equally little to both components. Thus, Dim1 could be linked with enzymes, sugars, TFC and chlorophyl—a broad range of variables and Dim2 with enzymes and phenols. In the group *R.s*. (Figure 8D) a largely scattered distribution can again be noted, with only CAT, SOD and POX, and GST, GR and APX clustered closely. Dim1 positively correlates with MDA, SS, negatively with CAR and TFC, while Dim2 correlates positively with GST, GR and APX and negatively with TPC and CHL, leaving POX and SOD, which contribute to both components, but slightly more so to Dim1. This makes naming the components difficult. Overall, data on family 41–65–7 K show inoculation with *P.p*. keep the distribution of the variables similar with the control group, while in the other two treated groups a clear scattering effect can be noted. Thus, a strain‐specific pattern appears.

**FIGURE 9:**
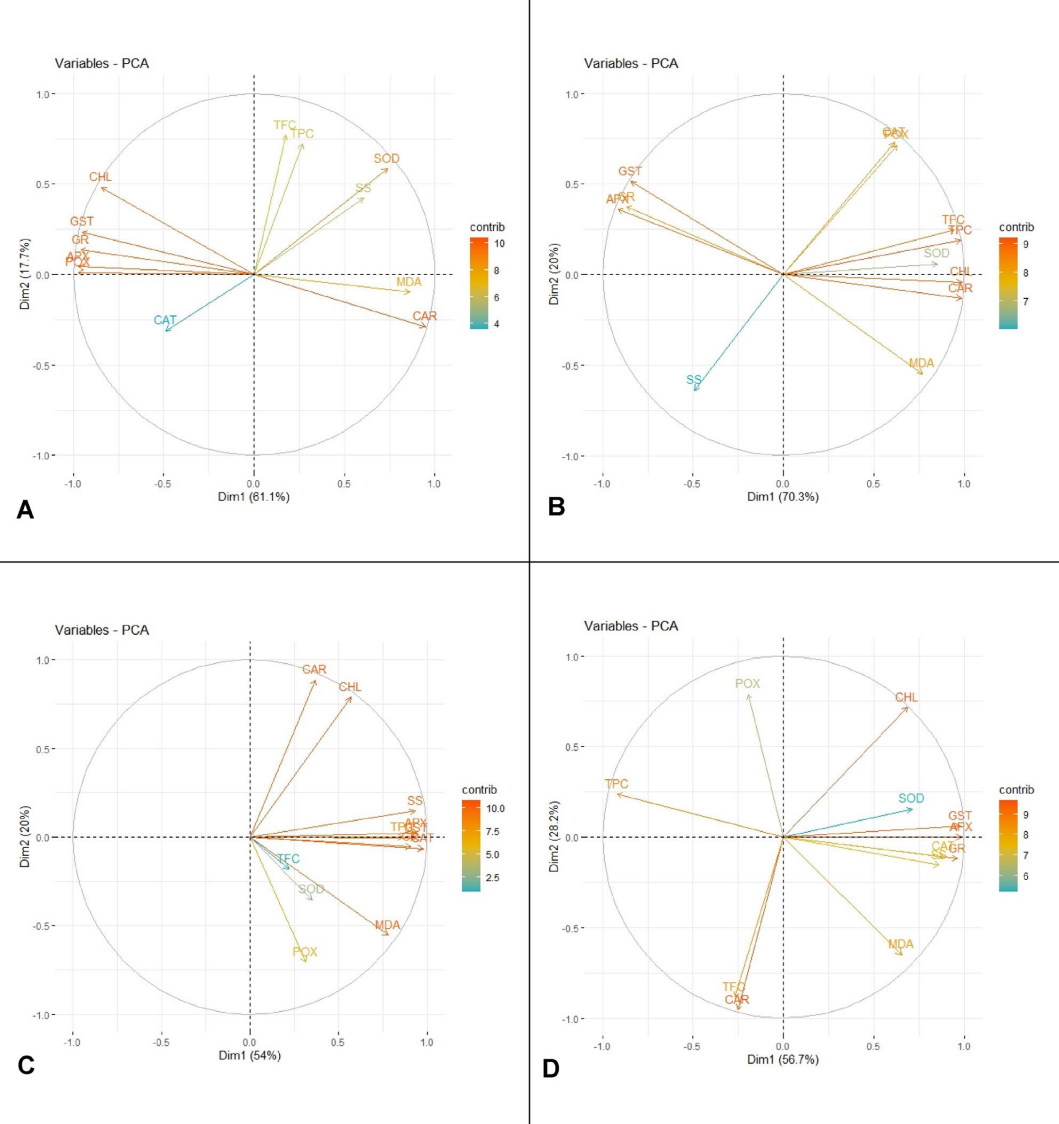
PCA results for phytochemical profile in black alder half‐sib family 13–99–1 K across different microbial treatments: Control (A), *Pseudomonas putida* (*P.p*.) (B), *Sphingobium yanoikuyae* (*S.y*.) (C), and *Rhodotorula sphaerocarpa* (*R.s*.) (D).

In all four groups the two principal components explain 74.0% to 90.3% of cumulative variance. In the control group (Figure 9A), Dim1 strongly positively correlates with MDA and CAR and negatively with CHL, GST, GR, APX and POX. CAT, SOD and SS correlate moderately with both components and TFC and TPC correlate positively with Dim2. This would be indicative that Dim2 can be associated with phenols, that is, non‐enzymatic antioxidant system and Dim1 with mainly antioxidant enzymes and oxidative damage, that is, enzymatic antioxidant systems. TFC and TPC are grouped together, as are GST, GR, APX and POX indicating strong links. The latter group is also linked with CHL. MDA and CAR are linked together, so are SOD and SS. In the *P.p*. group (Figure 9B) Dim1 strongly positively correlates with TFC, TPC, SOD, CHL, CAR and negatively with GST, GR and APX. SS, CAT, POX and MDA correlate moderately with both components but are more linked with Dim2. Dim1 could therefore be interpreted as primary and secondary metabolism and Dim2 as enzyme response. GST, APX and GR are again grouped close together, so are CAT and POX. TFC, TPC, SOD, CHL and CAR are clustered too, while SS and MDA stand apart. In the *S.y*. group (Figure 9C) Dim1 strongly positively correlates with SS, APX, TPC, GST, GR and CAT (grouped together), also moderately with MDA. Dim2 correlates positively with CAR and CHL (grouped together), and negatively with POX. Both TFC and SOD do not express any strong links with either principal component. This distribution could be identified as Dim1 being the secondary metabolites and Dim2 being the photosynthesis pigments. TFC, SOD, MDA and POX are also all clustered. In group *R.s*. (Figure 9D) Dim1 is positively correlated with GST, APX, CAT, GR, SS and SOD (grouped together), and negatively with TPC. Dim2 is positively correlated with POX, TFC and CAR (TFC and CAR grouped together), while MDA and CHL correlate similarly with both components. This distribution again indicates that Dim1 is more strongly linked with antioxidative enzymes, while Dim2 does not exhibit a strong trend either way. Overall, the data on family 13–99–1 K indicates that all treatments express a variety of effects on alder biochemistry. Notably, Dim1 is always linked with antioxidative enzymes, but this correlation generally shifts from strongly negative to positive in *S.y*. and *R.s* groups. According to results of PCA *P.p*. lightly modulates the scattering, while the latter two microorganisms change the biochemical relationships quite markedly. Also, in all treated groups MDA position in relation to Dim2 changes as well, indicating a multidimensional relationship.

In both genotypes a consistent strong grouping of most of the tested antioxidant enzymes generally remain in all of the tested treatments. This coincides with other results as well indicating their collective impact. However, in both genotypes a clear trend for this oxidative stress related group of variables to be impacted by the inoculation with the tested microorganisms can also be noted. Overall, the PCA data further reiterate both the alder genotype‐specific response and microorganism strain‐specific response.

### 3.2 Plant Growth‐Promoting Traits (PGPT) of Microorganisms

Results of the PGPT assessment showed that all three of the tested microorganisms exhibited various traits and were fundamentally different in this regard (Table 1). *P.p*. was able to produce siderophores and assimilate both forms of phosphate. Moreover, out of the tested three microorganisms it was capable of producing the largest relative amount of IAA within a given timeframe. Similarly, *R.s*. also produced siderophores, but other tests showed no significant results. *S.y*., on the other hand was the only tested microorganism that was able to growth on nitrogen‐free medium, however it did not distinguish itself in the other tests.

**TABLE 1:** Plant growth promoting trait assessment for the three tested microorganisms.

	Siderophore production	Potassium solubilisation	Nitrogen fixation	Indole‐3‐acetic acid production, μg/mL per 24 h	Biofilm production	Inorganic phosphate solubilisation	Organic phosphate mineralization
*Pseudomonas putida*	+	−	−	25.83	Weak	+	+
*Rhodotorula sphaerocarpa*	+	−	N/A	3.3	Weak	−	−
*Sphingobium yanoikuyae*	−	−	+	15.16	Weak	−	−

## 4 Discussion

Plant growth‐promoting bacteria have been extensively studied for their ability to enhance plant productivity. All three of the microorganism genera tested (*Rhodotorula*, *Sphingobium* and *Pseudomonas*) in this study have been found in association with forest soil or forest plants, thus their introduction into the forest ecosystems is unlikely to cause damage in terms of community biodiversity (Aghai et al. 2019; Allahkarami et al. 2021; Angeles de Paz et al. 2023; Costa‐Gutierrez et al. 2022; Frasson et al. 2017; Han et al. 2014; Haňáčková et al. 2017; Kandel, Joubert, and Doty 2017; Khan et al. 2016; Moller, Lerm, and Botha 2016; Zhao et al. 2002). Our study contributes to the growing body of research focusing on the innovative use of PAH‐degrading bacteria to enhance plant growth and health, particularly in the context of phytoremediation. Chaudhry et al. (2005), Ijaz et al. (2015), and Lladó et al. (2013) (Abd El‐wahed et al. 2023; Jou et al. 2022; Sultana et al. 2024; Weyens et al. 2010, 2012). According to the literature, strains of *Pseudomonas putida* (*P.p*.) and *Pseudomonas fluorescens* Migula 1895 have been particularly effective in promoting root and shoot elongation in crops such as canola, corn, lettuce and tomato, as well as enhancing yields in potato, radish, rice, sugar beet, apple, citrus, bean and wheat (Glick et al. 1997; Mehnaz and Lazarovits 2006; Molina‐Romero et al. 2017; Weyens et al. 2012; Xie, Pasternak, and Glick 1996). Furthermore, *P.p*. was evidenced to improve chickpea drought tolerance by enhancing seed germination, ROS scavenging ability, shoot length and dry weight. Inoculation also induced lower proline and MDA levels (both are stress indicators). It's noteworthy that a significant difference was noted between the two tested chickpea varieties, whereby one was far more favourably affected than the other (Tiwari et al. 2016), which is generally similar to the results of the current study. A few studies that have been conducted with trees using the aforementioned microorganism genera demonstrated promising results. Most notably perhaps, *P.p*. was shown to enhance poplar (*Populus* sp.) biomass and reduce trichlorethylene (TCE) toxicity simultaneously (Weyens et al. 2010). Later, the same strain was used to boost not only poplar biomass, but also was shown to lower SOD and GR levels (Weyens et al. 2012). Moreover, *P.p*. was also demonstrated to reduce naphthalene phytotoxicity by degrading it in the soil when peas were inoculated with it. Furthermore, plants treated with inoculant were exposed to naphthalene, their seed germination and transpiration rates were higher than those of the untreated controls. Additionally, the inoculated plants exhibited a 40% higher rate of naphthalene degradation in artificially contaminated soil compared to the untreated plants (Germaine et al. 2009).

Data show that a sphingomonad species, *Sphingopyxis panaciterrae* ( *S. panaciterrae* ) Lee et al. (2011), was recently proven to be an effective plant growth promoter in spinach, whereby it increased plant height, weight (up to 66% and 37% respectively), chlorophyll and carotenoid content (193% and 212% respectively), as well as antioxidant levels (increase up to 207%) in the aboveground biomass (Sultana et al. 2024). This corresponds with our results, reflecting its potential to enhance photosynthetic efficiency and stress resilience in black alder as well. Additionally, *Sphingobium* species have been documented to significantly boost the growth, fresh biomass, and chlorophyll content of different plants under diverse abiotic stress conditions (Khan et al. 2014, 2017; Luo et al. 2019). Increasing primary metabolite concentration and growth while reducing antioxidant enzyme concentrations may be attributed to the interaction between the microorganisms and black alder seedlings, which potentially lowers stress levels and enhances resilience. Moreover, *Sphingobium* sp. can use PAHs as a carbon source (Zylstra and Kim 1997) (Cunliffe and Kertesz 2006; Mitra et al. 2020), thus directly reducing phytotoxicity.

Furthermore, *Rhodotorula sp*. was shown to alleviate drought and *Fusarium* wilt‐induced stress in tomato plants. Alongside lower disease severity and disease incidence levels, the yeast induced enzyme, phenolic compounds and flavonoid production, and enhanced growth (increased biomass and height) (Abd El‐wahed et al. 2023). This is consistent with our findings, demonstrating the yeast's potential to enhance plant growth. Further analyses in the current study of the tested PAH‐degrading microorganisms revealed that they possess certain qualitative characteristics that may be associated with aforementioned plant growth promotion. For example, IAA production is often linked with increased root system parameters, as potentially was the case for *Arabidopsis thaliana* inoculated with *P.p* (Arslan and Akkaya 2020). Moreover, *S.y*. was also shown to induce IAA production in *Salix* trees (Zeng et al. 2020) and rice (Jou et al. 2022), while other *Sphingobium* spp. exhibited IAA production and nitrogen fixation capabilities (Sukweenadhi et al. 2015; Sultana et al. 2024). In all of these cases authors deduced that varied plant growth promoting traits may be the key reason behind plant growth and health improvement. Based on the evidence presented, it can be hypothesised that these PGPTs might interact to enhance plant growth and subsequently contaminant uptake. For example, nitrogen fixation, easier iron and phosphorus assimilation would increase nutrient availability, while hormone production would stimulate root growth, thus increasing overall biomass, allowing for more effective contaminant absorption. Similarly, enhanced resilience, which is indicated by enhanced growth and an active enzymatic and non‐enzymatic antioxidant systems (as expressed by phenols, enzymes, etc.) may also directly lead to optimised contaminant absorption.

## 5 Conclusions

This study underscores the potential of polycyclic aromatic hydrocarbon (PAH)‐degrading microorganisms in promoting the growth and health of black alder ( *Alnus glutinosa* ) seedlings. Noteworthy findings indicate that microbial inoculation, particularly with the yeast *Rhodotorula sphaerocarpa*, resulted in significant increases in shoot growth and root length within the 41–65–7 K half‐sib family, highlighting the family‐dependent variability in response to microbial treatments. Variations in the chlorophyll a/b ratio, stress indicator levels, and antioxidant enzyme activities further elucidate the nuanced effects of different microorganisms. The evaluation of plant growth‐promoting traits revealed that the tested microorganisms possess attributes that may support plant growth and enhance phytoremediation efficiency. These results highlight the potential for leveraging genetic diversity to optimise plant performance in PAH‐contaminated environments and suggest that the strategic use of selected PAH‐degrading microorganisms can significantly enhance phytoremediation efforts by promoting overall plant health and growth. It is important to note, however, that the findings from this study, conducted under controlled hydroponic conditions, must be contextualised for broader experimental applications.

## Author Contributions

**Greta Striganavičiūtė:** conceptualization, data curation, formal analysis, investigation, methodology, software, resources, visualization, writing – original draft, writing – review and editing, validation. **Dorotėja Vaitiekūnaitė:** conceptualization, data curation, formal analysis, investigation, methodology, visualization, writing – review and editing, validation. **Milana Šilanskienė:** data curation, investigation, visualization, writing – review and editing. **Vaida Sirgedaitė‐Šėžienė:** conceptualization, methodology, resources, supervision, writing – review and editing, validation.

## Conflicts of Interest

The authors declare no conflicts of interest.

## Data Availability

All data for this study are in the article and supplementary materials. Additional data can be requested from the corresponding author.
